# Sudden unexpected infant death, sudden unexplained death in childhood, and sudden unexpected death in epilepsy

**DOI:** 10.1111/dmcn.16226

**Published:** 2024-12-22

**Authors:** Suvasini Sharma, Robyn Whitney, Sayoni Roy Chowdhury, Rajesh Ramachandrannair

**Affiliations:** ^1^ Department of Pediatrics The Hospital for Sick Children Toronto Canada; ^2^ Department of Pediatrics McMaster University Hamilton Canada; ^3^ Department of Pediatrics Lady Hardinge Medical College and Kalawati Saran Children's Hospital New Delhi India

## Abstract

Sudden deaths in infants and children represent a profound and tragic event that continues to challenge researchers despite extensive investigation over several decades. The predominant phenotype, sudden infant death syndrome (SIDS), has evolved into the broader category of sudden unexpected infant death (SUID). In older children, a less understood phenomenon known as sudden unexplained death in childhood (SUDC) has garnered attention. Additionally, sudden unexpected death in epilepsy (SUDEP) constitutes a rare but recognized complication of epilepsy. Recent investigations indicate overlapping clinical, neuropathological, and genetic characteristics among SUID, SUDC, and SUDEP. Common features include death occurring during sleep, discovery in the prone position, hippocampal abnormalities, and genetic variations associated with epilepsy or cardiac arrhythmias. Notably, video recordings in certain examples of SUDC have captured ‘convulsive’ episodes preceding death in children without prior seizure history, suggesting that seizures may contribute more significantly to sudden paediatric deaths than previously presumed. This review explores these shared elements, underscoring their importance in formulating possible preventative measures against these devastating conditions.

**Abbreviations**
SIDS: sudden infant death syndrome
SUID: sudden unexpected infant death
SUDC: sudden unexplained death in childhood
SUDEP: sudden unexpected death in epilepsy

**What this paper adds**

- Sudden unexpected infant death, sudden unexplained death in childhood, and sudden unexpected death in epilepsy share clinical, neuropathological, and genetic features.
- Seizures might cause more sudden paediatric deaths than previously thought.

Sudden death in infants and children remains a devastating phenomenon that continues to pose significant challenges despite extensive research efforts spanning decades. The most widely recognized condition is sudden infant death syndrome (SIDS), now categorized under the broader term sudden unexpected infant death (SUID). In older children, sudden unexplained death in childhood (SUDC) represents a less studied but similarly tragic occurrence. Sudden unexpected death in epilepsy (SUDEP) is a rare event in paediatric epilepsy. A common characteristic across these conditions is their association with sleep. Traditionally, SUID and SUDC have not been widely linked to epilepsy; however, emerging evidence suggests potential shared underlying mechanisms among these entities.^1^ This review aims to synthesize recent findings exploring these overlaps. Understanding these connections is crucial for developing effective preventative strategies against these heartbreaking conditions.

## SUDDEN UNEXPECTED DEATH IN EPILEPSY

SUDEP represents the most prevalent epilepsy‐related cause of death among individuals living with epilepsy, encompassing both children and adults. SUDEP is characterized by the abrupt and unexpected nature of death, which can occur in either the presence or absence of witnesses. It is non‐traumatic and non‐drowning, and occurs in patients with epilepsy, with or without evidence of a seizure. Notably, SUDEP excludes cases of documented status epilepticus, and postmortem examination does not reveal any toxicological or anatomical causes of death.^2^ Although once believed to be rare in childhood, the incidence of SUDEP in children and adults is similar: approximately 1.2 per 1000 person years.^3^, ^4^, ^5^

The most critical risk factor for SUDEP is the occurrence of bilateral tonic–clonic seizures, previously called generalized tonic–clonic seizures, especially if frequent and uncontrolled.^6^ Nocturnal bilateral tonic–clonic seizure is the second most important risk factor. Other risk factors for SUDEP include living alone, a lack of seizure freedom in the preceding 1 to 5 years, and medication non‐adherence.^6^ Certain genetic epilepsy disorders are also at increased risk of SUDEP (i.e. Dravet syndrome). The pathophysiology of SUDEP is not well understood but may involve mechanisms such as cerebral shutdown, autonomic dysfunction, altered brainstem function, and cardiorespiratory shutdown.^7^ A triggering bilateral tonic–clonic seizure may lead to preterminal events comprising cardiac, respiratory, autonomic, and cerebral dysfunction.^7^ However, SUDEP deaths may also occur without any evidence of recent seizure activity, and the pathophysiology in these cases is largely unknown. SUDEP prevention strategies include reducing the frequency of tonic–clonic seizure, improving compliance, avoiding seizure triggers (i.e. sleep deprivation), and the use of nocturnal supervision and/or listening devices.^8^

## SUDDEN UNEXPECTED INFANT DEATH AND SUDDEN INFANT DEATH SYNDROME

SUID is defined as any sudden and unexpected death, whether explained or unexplained, occurring during infancy.^9^ The term SIDS is used for deaths of infants younger than 1 year of age that remain unexplained after investigation, autopsy, medical history review, and appropriate laboratory testing.^9^ SIDS is a leading cause of infant deaths in high‐income countries.^10^ Among SUID cases, more than 90% occur in the first 6 months of life, peaking at 2 to 4 months.^11^ The most well‐known risk factor for SIDS is sleeping prone.^11^ Other sleep‐related risk factors include soft bedding and bed‐sharing. Preterm birth, low birthweight, prenatal and postnatal smoke exposure, maternal alcohol and illicit drug use, and unsafe sleep environments are other clinical risk factors for SIDS.^10^

The most well‐described and accepted pathogenic framework of the pathomechanism of SIDS is the triple risk model, which describes a combination of: (1) exogenous factors or stressors (e.g. prone or side sleep position, overbundling, airway obstruction); (2) a critical vulnerable period of development (2–4 months of age); and (3) intrinsic vulnerability (e.g. dysfunctional and/or developing cardiorespiratory and/or arousal systems) leading to death.^10^, ^12^

## SUDDEN UNEXPLAINED DEATH IN CHILDHOOD

SUDC is the term used to describe a death without a determined cause in a child aged 1 to 18 years old.^13^ SUDC is more common in males aged 1 to 4 years.^13^ Children who die of SUDC are often found in a prone position.^14^ Fever or illness has been commonly noted in the 72 hours preceding death in both SUDC and SUID.^14^, ^15^ In an extensive series of 151 sudden deaths in young children, 121 of whom were SUDC, almost half (48.8%) had a personal and/or family history of febrile seizures.^15^

## COMMON THEMES BETWEEN SUDEP, SUID, AND SUDC

**FIGURE 1:**
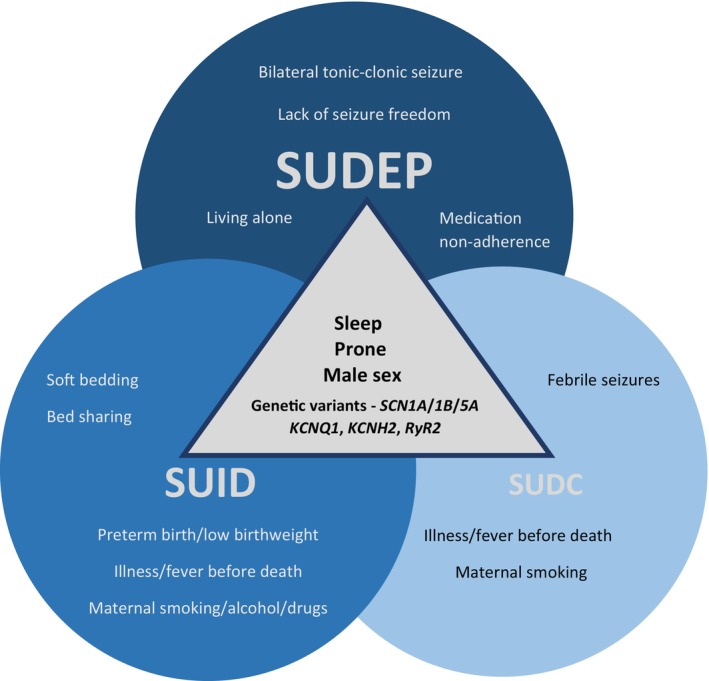
Risk variables for seizure‐related deaths in children: sudden unexpected death in epilepsy (SUDEP), sudden unexpected infant death (SUID), and sudden unexplained death in childhood (SUDC)—a continuum.

SUID, SUDC, and SIDS share certain clinical, neuropathological, and genetic features (Figure 1). The typical clinical aspects include male predominance, unwitnessed deaths, death during sleep, and discovery in the prone position.^1^ Hippocampal anomalies on neuropathology, suggesting the possibility of underlying epilepsy, have been identified in sudden explained child deaths.^15^, ^16^ A strong past or family history of febrile seizures has been noted in children with SUDC.^17^ As described later, there is a likelihood that a small subset of children with SUID and SUDC had seizures preceding death.^18^ Lastly, pathogenic variants in epilepsy and cardiac arrhythmia genes have been identified in cases of SUDC and SIDS, again suggesting an overlap with SUDEP.^19^ These common features suggest that a subset of unexpected deaths in infants and children result from the possibility of unrecognized seizures.

## EVIDENCE OF SEIZURES IN SUDC AND SUID

The landmark MORTEMUS study retrospectively analysed video‐electroencephalogram (EEG) data to study the occurrence of SUDEP in epilepsy monitoring units.^20^ The sequence of events postulated is that a tonic–clonic seizure in sleep induces an early postictal, centrally mediated, severe cardiorespiratory dysfunction, which either results in death immediately or leads to a transient period of partly restored cardiorespiratory function followed by terminal apnoea and then cardiac arrest.^20^

Similar ‘convulsive’ events in sleep preceding death have been noted in some cases of SUID and SUDC. One premorbidly typically developing 8‐month‐old male infant was discovered seizing while sleeping in the prone position, brought to the hospital where seizures were diagnosed, and declared brain dead 2 days later with the withdrawal of life support.^21^ The authors postulated that this event could be a cardiorespiratory SIDS event with a primary brainstem pathology or the first manifestation of an underlying inborn error of metabolism presenting as a fasting‐induced seizure, or SUDEP, with the triggering seizure being the first manifestation of an underlying epilepsy.^21^

Myers et al. reported the case of a 20‐month‐old female with a chromosomal disorder who developed febrile status epilepticus and was subsequently admitted and started on video‐EEG monitoring.^22^ She died suddenly after 2 days while on video‐EEG monitoring. Diffuse cerebral suppression on EEG was observed 10 minutes before death, followed minutes later by severe bradycardia.^22^ These findings were similar to those seen in SUDEP. Recently, Gould et al. reported the analysis of home video recordings of seven toddlers with SUDC of the children's last sleep period preceding death.^18^ Six out of seven children experienced a ‘convulsive’ event preceding death. Six were prone with face down, and one had autopsy evidence of airway obstruction. Out of these, only one child had a prior history of febrile seizures. There was no evidence of cardiac pathology in the autopsies of these children. Also, whole exome sequencing did not reveal any cardiac disease variants.^18^ This study provides striking evidence of similarities between SUDEP and SUDC and suggests that seizures in sleep may be the inciting event for SUDC.

Seizures may also be responsible for some cases of sudden infant deaths, which are usually unwitnessed during sleep. Infant seizures are often subtle and may cause isolated apnoeas, and hence may be missed before the terminal event.^18^ In a study of 17 infants with apparent life‐threatening events and focal seizures with normal interictal EEGs, six infants had ictal EEG abnormalities followed by tachycardia, oxygen desaturations, and apnoeas, which persisted despite resuscitation.^23^ This study demonstrated that epileptic seizures can potentially be associated with life‐threatening apnoeas.^23^ This subset of SUID cases mimics the sequence in SUDEP in which a seizure provokes respiratory abnormalities and/or cardiac rhythm disturbances.

However, it needs to be emphasized that unrecognized seizures preceding death in SUID possibly represent a small subset of children with SUID. Ictal apnoeas are rare, even in neonates. This mechanism for SUID must still be considered speculative, pending further evidence.

## FEBRILE SEIZURES AND SUDC

Febrile seizures occur in 2% to 5% of children between the ages of 6 months and 6 years, but approximately one‐third of SUDC cases have a history of febrile seizures, and around half have a personal or family history of febrile seizures.^15^, ^24^ Among SUDC cases 1 to 6 years of age, 28% had a febrile seizure history compared to less than 3% of typically developing peers at age 1 year 7 months (median age of SUDC).^25^ Interestingly, although simple febrile seizures are considered benign, in a systematic review comparing rates of mortality between children with simple and complex febrile seizures and typically developing peers, 78% (104/133) of deaths with a history of any febrile seizure type had only simple febrile seizures. These results may have an implication in parental counselling of children with simple febrile seizures, emphasizing that though these are by and large benign, there is a very rare risk of sudden death, and potential prevention strategies such as sleep supervision can be explored.^26^ An autosomal dominant pattern of febrile seizure inheritance has been noted in families with SUDC and febrile seizures, but further studies are needed to identify genetic variants which cause children with simple febrile seizures to be at risk for sudden death.^17^

## HISTOPATHOLOGICAL SIMILARITIES

Neuropathological studies have revealed findings suggestive of underlying epilepsy in some cases of SUID and SUDC in children, even without a prior history of epilepsy or seizures.^27^ These abnormalities have been termed ‘epilepsy in situ’.^15^, ^28^ The commonly reported abnormalities are in the hippocampus, which is implicated in up to 40% to 60% of SUID, SUDC, and SUDEP.^1^, ^16^ The most frequent hippocampal abnormality in sudden death spanning the age spectrums of both SIDS and SUDC was dentate gyrus lamination, which denotes the presence of focal double layering in the otherwise single layer of granule cells in the dentate gyrus.^29^ This is a finding reported in temporal lobe epilepsy^30^ and has also been noted in adult cases of temporal lobe epilepsy. Dentate gyrus abnormalities have also been reported in SUDEP.^31^ Other hippocampal abnormalities (hippocampal malrotation, hippocampal focal malformation, and hippocampal formation maldevelopment) have also been reported in SUDC.^27^ It is still not clear whether these abnormalities are the cause or result of seizures, but many children with SUDC and hippocampal abnormalities had no history of seizures.

## SIMILARITIES IN MOLECULAR GENETICS

Several genes may contribute to epilepsy and sudden unexpected death in children and adults, with specific variants associated with SUDEP, SUDC, and SUID. Pathological variants in genes encoding voltage‐gated sodium channels have been linked to neurological and cardiac disorders, and have been implicated in SUDEP, SUID, and SUDC.^1^ Genetic risk factors have been identified on postmortem testing in 30% to 50% of patients with SUDEP; however, the majority of these patients have a monogenic condition and SUDEP, where the monogenic condition predisposes to SUDEP.^32^ In a sizeable exome‐based analysis of cardiac arrhythmia, epilepsy, and respiratory control genes in patients with SUDEP, pathogenic or candidate pathogenic variants were identified in 46%.^33^ These included pathogenic variants in long QT syndrome genes (i.e. *SCN5A, KCNH2, KCNQ1*) in 7%, genes predisposing to malignant cardiac arrhythmia (i.e. *ANK2, AKAP9, HCN4*) in 15%, and epilepsy‐related genes were found in 25% of the cases.^33^ In a recent genetic testing study of 39 SUDEP patients, gene panels detected 62 unique variants in 45 genes: 19 on the arrhythmia panel and 26 on the epilepsy panel.^34^

Pathogenic variants in epilepsy and cardiac arrhythmia genes have also been identified in cases of SUDC and SIDS. In a whole exome sequencing analysis of 73 cases of paediatric sudden unexpected death, pathogenic variants were found in 11 brain/cardiac voltage‐gated sodium channel genes (*SCN1A*, *SCN3A*, *SCN4A*, *SCN9A*, *SCN10A*, *SCN1B*).^19^ Interestingly, out of the 11 patients with pathogenic variants in the voltage‐gated sodium channel genes, seven had hippocampal abnormalities on neuropathology, and one had a history of febrile seizures.^19^ However, many children who die of SUID and SIDS and harbour pathogenic variants in voltage‐gated sodium channel genes and dentate gyrus lesions do not have a history of seizures or epilepsy, indicating that these children may have had unrecognized or unwitnessed seizures in the past.^1^

In a recent large study of genetic contributions to sudden unexpected death in paediatrics (including both SUDC and SUID cases), exome sequencing identified contributory variants in 11% of the cases, including pathogenic variants in epilepsy and cardiac genes such as *SCN1A, DEPDC5, GABRG2, SCN5A, TTN*, *MYBPC3, PLN*, and *TNNI3*.^35^ Genetic analysis of 84 cases with dentate gyrus bilamination in this series revealed genetic abnormalities in 10 cases comprising variants in genes associated with neurological disease (*DEPDC5, GABRB3, GABRG2*), cardiac disease (*AKAP10, SCN5A, TNNI3, TTN*), and two in genes associated with systemic/syndromic disease (*FLNA, TCF4*).^35^

## CONCLUSION

SUID, SUDC, and SUDEP share clinical, neuropathological, and genetic features, including male predominance, unwitnessed deaths, death during sleep, discovery in the prone position, hippocampal abnormalities, and variants in genes associated with epilepsy and cardiac rhythm disturbances. A predisposition towards febrile seizures or epileptic seizures could cause death directly from seizure‐associated apnoea with or without cardiac rhythm disturbances. Seizures may be a more frequent cause of sudden paediatric deaths than previously thought. This area needs more research so that high‐risk children can be identified for appropriate parental cou and preventative strategies, such as nocturnal supervision, may be considered.

## Data Availability

Not applicable.
